# Caudal cervical vertebral morphological variation is not associated with clinical signs in Warmblood horses

**DOI:** 10.1111/evj.13140

**Published:** 2019-07-16

**Authors:** S. Veraa, K. de Graaf, I. D. Wijnberg, W. Back, H. Vernooij, M. Nielen, A.J. M. Belt

**Affiliations:** ^1^ Division of Diagnostic Imaging, Faculty of Veterinary Medicine Utrecht University Utrecht the Netherlands; ^2^ Equine Clinic Veterinary Centre Someren Someren the Netherlands; ^3^ Department of Equine Sciences, Faculty of Veterinary Medicine Utrecht University Utrecht the Netherlands; ^4^ Department of Surgery and Anaesthesiology of Domestic Animals, Faculty of Veterinary Medicine Ghent University Merelbeke Belgium; ^5^ Department of Farm Animal Health Utrecht the Netherlands

**Keywords:** horse, anatomy, transitional vertebra, homologous

## Abstract

**Background:**

Variation in equine caudal cervical spine morphology at C6 and C7 has high prevalence in Warmblood horses and is suspected to be associated with pain in a large mixed‐breed group of horses. At present no data exist on the relationship between radiographic phenotype and clinical presentation in Warmblood horses in a case‐control study.

**Objectives:**

To establish the frequency of radiographically visible morphologic variation in a large group of Warmblood horses with clinical signs and compare this with a group without clinical signs. We hypothesised that occurrence of morphologic variation in the case group would not differ from the control group, indicating there is no association between clinical signs and morphologic variation.

**Study design:**

Retrospective case‐control.

**Methods:**

Radiographic presence or absence of morphologic variation of cervical vertebrae C6 and C7 was recorded in case (n = 245) and control horses (n = 132). Case and control groups were compared by univariable Pearson’s Chi‐square and multivariable logistic regression for measurement variables age, sex, breed, degenerative joint disease and morphologic variation at C6 and C7. Odds ratio and confidence intervals were obtained. A P≤0.05 was considered statistically significant.

**Results:**

Morphologic variation at C6 and C7 (n = 108/377 = 28.6%; Cases 58/245 = 23.7%; Control 50/132 = 38%) was less frequent in horses with clinical signs in univariable testing (OR 0.48, 95% CI 0.3–0.8, P* *=* *0.001). Age, sex, breed and degenerative joint disease were not retained in the final multivariable logistic regression step whereas morphologic variation remained significantly less present in horses with clinical signs.

**Main limitations:**

Possible demographic differences between equine clinics.

**Conclusions:**

Morphologic variation in the caudal cervical spine was detected more frequently in horses without clinical signs. Therefore, radiographic presence of such variation does not necessarily implicate the presence of clinical signs.

## Introduction

The occurrence of vertebral morphologic variations in the equine cervico‐thoracic vertebral column, such as rudimentary first ribs and shape variations of C7, has been described as early as the beginning of the 20th century, even before radiography was performed in horses 1, 2. At that time, it was believed that a decrease in the number of ribs was a sign of evolutionary progression whereas an increase would indicate evolutionary devolution 1.

About a century later, the role of the *Hox*‐genes in defining cervical vertebral numbers and shape was established 3. Variations in cervical vertebral numbers (homeotic changes) have been reported in horses as occipito‐atlanto‐axial malformation (OAAM) 4, 5, 6, and also as the presence of rudimentary or malformed ribs which seem to coincide in all cases with shape variations of C6 and C7 7, 8, 9. Existence of vertebral morphologic variation in the caudal cervical spine has been reported in one‐third of both Thoroughbred and Warmblood populations 7, 10. This variation has been evaluated in more detail in radiographic studies in larger mixed groups of horses 11, 12. In their retrospective study DeRouen *et al*. 12 concluded that anomalous C6 vertebrae were more common in the Warmblood horses (n = 55, 79.2%), than in other breeds (n = 45, 47.4%) and possible relationships with cervical pain due to altered regional biomechanics were suggested. Vertebral morphologic variation has been reported as unilateral or bilateral and always in two consecutive (mostly C6 and C7) vertebrae 7, 11, 12.

Radiography remains the first and most important tool for screening and diagnosis of abnormalities of the equine cervical spine. It is important to assess the potential of this tool to distinguish between morphological variations that may or may not be associated with clinical signs. Therefore, the objectives of this retrospective case‐control study were to 1) establish the frequency of morphologic variation in cervical vertebrae of adult Warmblood horses with and without clinical signs related to the neck, and 2) to determine a possible association between variation at C6 and C7 with the presence of clinical signs.

## Materials and methods

### Study data

Study data, including cervical radiographic examinations, breed, age, sex, electromyography, clinical signs, when applicable, were obtained from the Division of Diagnostic Imaging of the Faculty of Veterinary Medicine, Utrecht University and the private equine clinic of the Veterinary Centre Someren.

Cervical radiographic examinations of all Warmblood horses (with and without clinical signs) performed between January 2011 and December 2013 (n = 245), were retrieved from the Picture Archiving and Communication System (Impax, version 6.6.1.5003)^1^ of the Division of Diagnostic Imaging of the Faculty of Veterinary Medicine, Utrecht University. Data of radiographic examinations of Warmblood horses without clinical signs, which had been performed during pre‐purchase examinations between January 2009 and December 2012 (n* *=* *132), were retrieved from the Picture Archiving and Communication System (EasyIMAGE, VetZ)^2^ of the equine clinic of the Veterinary Centre Someren. Radiographic examinations were excluded if only the cranial cervical spine was assessable.

The radiographic data were reviewed by one board certified radiologist (S.V.) who was not blinded to the clinical data for the presence (unilateral, bilateral) or absence of morphologic variation (absence of a ventral laminar part of the transverse process in combination with a ventral protuberance at another transverse process) and its anatomic location. Furthermore, presence or absence of degenerative joint disease of the articular facet joint of C6 and C7 was recorded.

The clinical data of the above‐mentioned horses were reviewed and considered to be relevant if classified in one of the following groups: spinal ataxia during neurologic examination, restricted (latero‐ or dorso‐) flexion of the neck, cervical pain on palpation, abnormal behaviour (e.g. head shaking, bolting), cervical muscle atrophy, lameness and more specifically presumed thoracic neurological lameness, and hypermetric gait of fore‐ or hindlimbs. Presumed brachial plexus neurologic lameness was defined as any lameness that did not alter after diagnostic anaesthesia and/or was diagnosed as such based on electromyography. Clinical examinations were performed by board certified surgeons and internal medicine specialists, as well as residents under their supervision.

### Data analysis

Measurement variables were presented as categorical data and median and range were calculated for age of case and control groups. Age was classified as younger and older horses, defined as 0–10 years old and >10 years old. Sex was defined as female and male. Breed was divided into Dutch Warmblood and other Warmbloods. Degenerative joint disease was defined as absent or present.

Attribute: Morphologic variation was first defined as present or not present; thereafter unilateral or bilateral variation were separated.

The following case definitions of the case group were used: A1) all horses with at least one recorded clinical sign, A2) horses with spinal ataxia, A3) horses with cervical pain on palpation, A4) horses with lameness. Clinical signs in case definitions A2 to A4 were considered as inclusion criteria but not as limiting factors. Therefore, combinations of the above mentioned recorded clinical signs were possible as horses could have more than one clinical sign recorded (see Table 1). In case definitions A1 to A3 presumed brachial plexus neurological lameness could be one of the recorded clinical signs; in case definition A4 all lameness cases were included.

**Table 1:** Distribution of clinical signs in absolute numbers and percentages for case groups (n = 245, n = 123, n = 106) and the control group (n = 132) with all horses included. Multiple clinical signs can be present per individual horse in a particular case group

Clinical signs		All (A1) (n = 245) n (%)	Spinal ataxia (A2) (n = 123) n (%)	Pain palpation (A3) (n = 106) n (%)	Lameness (A4) (n = 116) n (%)	Control (n = 132) n (%)
Spinal ataxia	Yes	123 (50.2)	**123 (100.0)**	61 (57.5)	40 (34.5)	0 (0)
	No	122 (49.8)	0 (0)	45 (42.5)	76 (65.5)	132 (100)
Restricted flexion of the neck	Yes	155 (63.3)	71 (57.7)	85 (80.2)	78 (67.2)	0 (0)
	No	90 (36.7)	52 (42.3)	21 (19.8)	38 (32.8)	132 (100)
Pain on palpation of the neck	Yes	106 (43.3)	61 (49.6)	**106 (100.0)**	51 (44.0)	0 (0)
	No	139 (56.7)	62 (50.4)	0 (0)	65 (56.0)	132 (100)
Abnormal behaviour (e.g. head shaking, bolting)	Yes	37 (15.1)	11 (8.9)	16 (15.1)	16 (13.8)	0 (0)
	No	208 (84.9)	112 (91.1)	90 (84.9)	100 (86.2)	132 (100)
Brachial plexus neurogenic lameness	Yes	27 (11.0)	12 (9.8)	15 (14.2)	27 (23.3)	0 (0)
	No	218 (89.0)	111 (90.2)	91 (85.8)	89 (76.7)	132 (100)
Muscular atrophy of the neck	Yes	46 (18.8)	24 (19.5)	22 (20.8)	19 (16.4)	0 (0)
	No	199 (81.2)	99 (80.5)	84 (79.2)	97 (83.6)	132 (100)
Hypermetria (fore‐ and/or hindlimbs)	Yes	32 (13.1)	22 (17.9)	15 (14.2)	14 (12.1)	0 (0)
	No	213 (86.9)	101 (82.1)	91 (85.8)	102 (87.9)	132 (100.0)

Note

The numbers in bold in case group A2 and A3, representing the case group selection criteria.

Associations between horse measurement variable (age, sex, breed and degenerative joint disease at C6 and C7), the attribute morphologic variation and groups (case‐control) were tested in two stages. The first stage included univariable analysis of the case‐control groups with each of the horse measurement variables and the attribute morphologic variations (yes/none, or unilateral/bilateral/none) by applying Pearson’s chi‐square test for all four case definitions. The odds ratios (OR) with 95% confidence interval were calculated, where the H_0_ for the OR = 1. Secondly, multi‐variable stepwise logistic regression analysis with backward elimination approach of the case‐control groups was performed in a subset of the horse study population including only those under 16 years of age and being Dutch Warmbloods, as measurement variables “age” and “breed” were seen to be most related to case‐control status during univariable analysis. Through this restriction, the case and control groups had a similar distribution for age and breed. Multivariable step‐wise logistic regression, with backward elimination approach, was performed for the respective four case definitions including age, sex, degenerative joint disease as measurement variables and unilateral/bilateral/none shape variation as attribute. Inclusion of a measurement variable in the four case‐control models depended on P<0.05. An eliminated variable was considered a confounding factor if it altered the coefficient (β) of the remaining variables by >10%. The odds ratios (OR) with 95% confidence intervals were calculated with OR = 1 considered according to the stated lack of difference between the two groups.

Statistical processing was carried out in SPSS (Version 24.0)^3^ (S.V.). A P≤0.05 was considered statistically significant.

## Results

### Horses

Included were 377 horses with radiographic examinations of the equine cervical spine and clinical recordings that met the requirements of this study. The frequency distribution of clinical signs for all case definitions is given in Table 1. Evaluated were 245 case horses (median age of 8 years; range 1–27 years; 146 geldings, 11 stallions and 87 mares and one unknown; 210 Royal Dutch Sporthorse, 9 Dutch Riding Horse 4 Belgian Warmblood, 6 Hanoverian, 3 Oldenburg, 5 Westfalen, one Holstein, 4 Zangersheide, one Rheinlander, one Trakehner, one Warmblood cross) and 132 control horses (median age 7 years; range 2–13 years; 71 geldings, 21 stallions and 40 mares; 82 Royal Dutch Sporthorse, one Dutch Riding Horse, 11 Belgian Warmblood, 8 Hanoverian, 11 Oldenburg, 2 Westfalen, 5 Holstein, 2 Zangersheide, 3 Rheinlander, 3 Polish Warmblood, one Trakehner, one Selle Français, one Swedish Warmblood, one Italian Warmblood) (see also Table 2).

**Table 2:** Frequency of variables in absolute numbers and percentages, odds ratio (OR), 95% confidence interval (CI) and P‐value of univariable Pearsons’ Chi‐square test between all horses with (case; n = 245) and without (control; n = 132) clinical signs

Variables	Categories	Case A1 (n = 245) n (%)	Control (n = 132) n (%)	OR A1	95% CI	P*‐*value A1
Age	NA	3 (1.2)	1 (0.8)			
	0–10	189 (77.1)	113 (85.6)	0.6	0.3–1.02	0.06
	>10	53 (21.6)	18 (13.6)	Ref.		
Sex	NA	1 (0.4)	0 (0)			
	Female	87 (35.5)	40 (30.3)	1.3	0.8–2.0	0.295
	Male	157 (64.1)	92 (69.7)	Ref.		
Breed	Dutch WB	210 (85.7)	82 (62.1)	3.7	2.2–6.0	0.0001
	Other	35 (14.3)	50 (37.9)	Ref.		
Degenerative joint disease	Absent	206 (84.1)	109 (82.6)	Ref.		
	Present	39 (15.9)	23 (17.4)	1.1	0.6–1.96	0.71
Morphologic variation C6–C7	None	190 (77.6)	82 (62.1)	Ref.		
	Yes	55 (22.4)	50 (37.9)	0.5	0.3–0.8	0.001
Morphologic variation C6–C7	None	190 (77.6)	82 (62.1)	Ref.		
	Unilateral	26 (10.6)	22 (16.7)	0.5	0.2–0.95	0.03
	Bilateral	29 (11.8)	28 (21.2)	0.5	0.3–0.8	0.06

NA, not available and not included in statistical results; Ref., reference group for odds ratio calculation; Dutch WB, Dutch Warmblood.

### Descriptive data analysis

**Figure 1:**
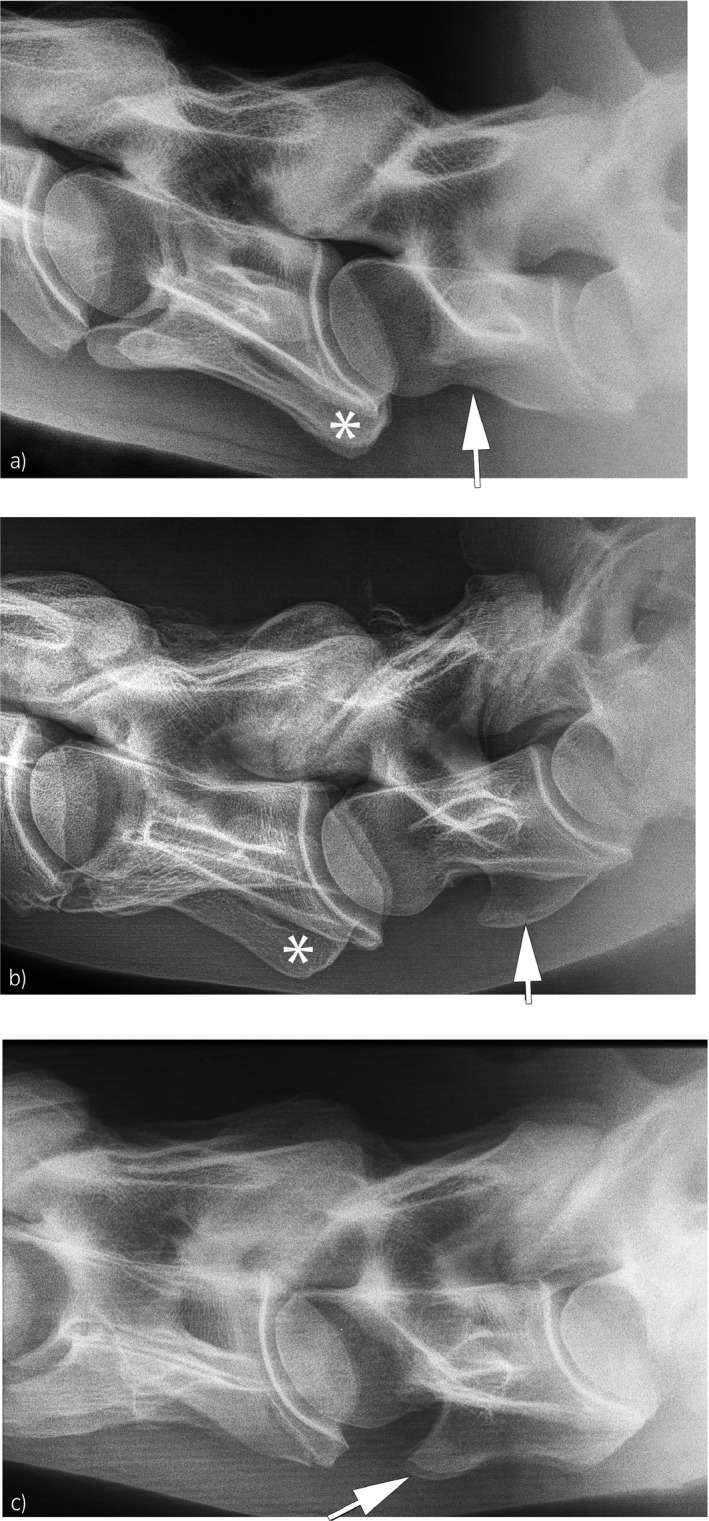
Morphologic variation of the caudal cervical spine; white asterisk marks the ventral laminar part of the transverse process of C6 and the black arrow marks the transverse process of C7. a) No variation is visible at C6 and C7 with a normal caudal part of the transverse process of C6 (asterisk) and horizontally oriented transverse processes of C7 (white arrow); b) unilateral variation is visible with only one caudal part of a transverse process of C6 (asterisk) and a single ventrally oriented protuberance at C7 (white arrow); c) bilateral variation is visible with no caudal part of the transverse process of C6 and two ventrally oriented protuberances at C7 (white arrow) as is arthrosis of the facet joints of C6–C7.

**Figure 2:**
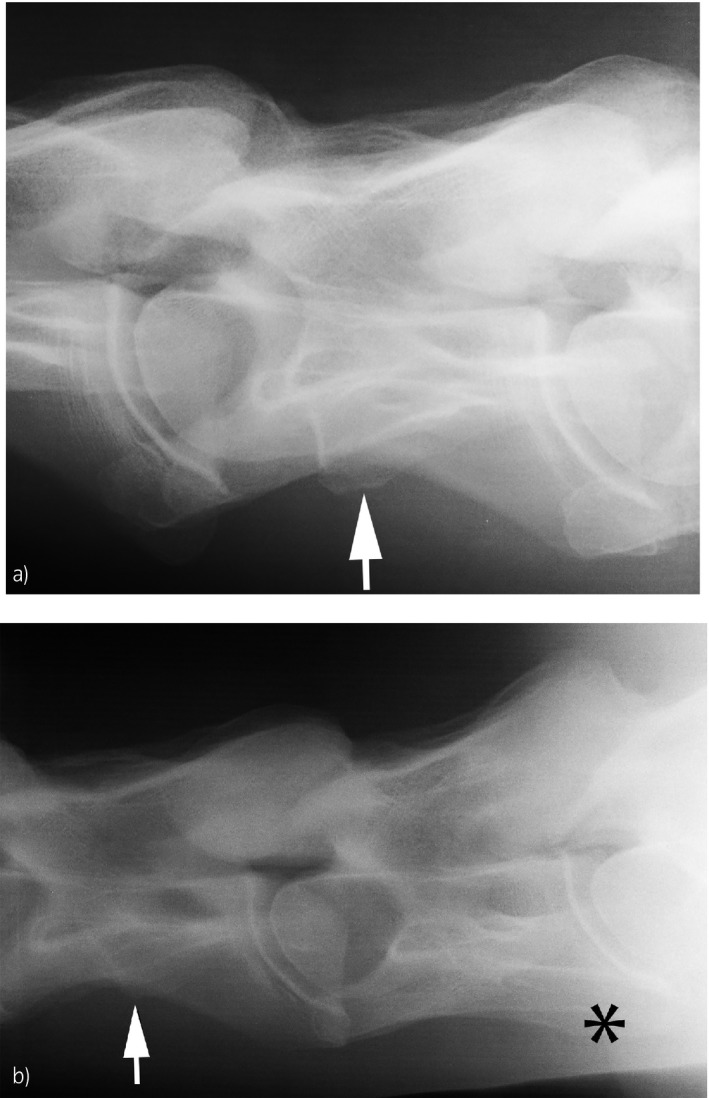
Morphologic variation at C5 and C6 is present. a) Note the ventrally protruding part of the transverse process at C5 (white arrow) and b) the unilateral transverse process of C6 (black asterisk).

Morphologic variation at C6 and C7 (case 58/245 = 23.7%; control 50/132 = 38.0%) consisted of absence of the ventral laminar part of the transverse process of C6 in combination with a ventral protuberance at the transverse process of C7 (Fig 1). This anomaly was seen unilaterally (n = 50) or bilaterally (n = 58). A single horse in the case group had a unilateral ventral protuberance at the transverse process of C5 with absence of the ventral part of the transverse process of C6 and no visible changes of C7 (Fig 2).

Location of lameness was recorded as follows: left front (n = 28), right front (n = 34), left hind (n = 3), right hind (n = 1), left front and right front (n = 39), left hind and right hind (n = 2), left front and right hind (n = 3), right front and left hind (n = 1), all limbs (n = 5).

Degenerative joint disease was scored as absent in 314 horses (206 in the group with and 108 in the group without clinical signs) and present in 63 horses (39 in the group with and 24 in the group without clinical signs).

Electromyography results were available and abnormal in 24 horses, including horses with a range of clinical signs, but most frequently showing signs of upper motor neuron involvement, e.g. signs of spinal ataxia (n = 8).

### Case‐control data analysis

#### Univariable analysis

A summary of the results of the univariable analysis with all horses included (i.e. A1) is shown in Table 2. For case definitions A2 to A4 and for a subset of horses <16 years old and Dutch Warmblood with case definitions A1 to A4, a summary of results is available in Supplementary Items 1 and 2.

When including horses of all ages and breeds, a higher age was noted in all case groups except for horses with spinal ataxia which were of similar age compared to the control group. There were more Dutch Warmblood horses in the case groups than the control group (P<0.05). There were no differences related to sex or degenerative joint disease between the two groups. Presence of morphologic variation at C6 and C7 was statistically significantly more in the control group for all case definitions (P<0.05). However, when divided in unilateral and bilateral morphologic variation compared to no variation, this was not true for all case definitions. The OR remained <1 for case horses for all case definitions for bilateral morphologic variations and only in case definition A1 for unilateral morphologic variation. For case definitions A2 to A4, the upper limit of 95% CI was >1 for cases.

#### Multivariable analysis

As variables age and breed were statistically different between case and control groups in univariable analysis, multivariable analysis was performed in a subset of horses <16 years old and Dutch Warmblood with case definitions A1 to A4. Decision making was based on the absence of horses older than 15 years of age in the control group and the overrepresentation of Dutch Warmblood horses in the case group. A summary of the results of multivariable analysis in the subset of horses being under 16 years old and Dutch Warmblood with spinal ataxia, is shown in Table 3. All results of other case definitions (all, pain on palpation, lameness) are included in Supplementary Item 3.

**Table 3:** Results of multivariable stepwise logistic regression analysis with backward elimination approach in a subset of horses, (<16 years of age and Dutch Warmblood), with morphologic variation divided in categories none‐unilateral‐bilateral; for cases defined as horses with spinal ataxia (n = 106) and the control group (n = 82)

Variable	P‐values	OR	95% CI
Step 1 (initial full model)			
Age (>10, <10)	0.13	0.5	0.2–1.2
Sex (male, female)	0.66	0.9	0.5–1.6
Degenerative joint disease (Yes, No)	0.87	1.1	0.4–2.6
Morphologic variation			
None	0.036		
Unilateral	0.025	0.4	0.2–0.9
Bilateral	0.65	0.8	0.3–2.4
Step 2			
Age	0.13	0.5	0.2–1.2
Sex	0.65	0.9	0.5–1.6
Morphologic variation			
None	0.035		
Unilateral	0.022	0.4	0.2–0.9
Bilateral	0.61	0.8	0.3–2.3
Step 3			
Age	0.13	0.5	0.2–1.2
Morphologic variation			
None	0.036		
Unilateral	0.023	0.4	0.2–0.9
Bilateral	0.62	0.8	0.3–2.3
Step 4 (Final reduced model)			
Morphologic variation			
None	0.054		
Unilateral	0.038	0.4	0.2–0.95
Bilateral	0.73	0.8	0.3–2.5

OR, odds ratio; 95% CI, 95% confidence interval.

Stepwise logistic regression analysis with backward elimination approach in the above mentioned subset of horses and division of morphologic variation (none‐yes) for all case definitions revealed that “morphologic variation” remained in the model but not the measurement variables sex, age and degenerative joint disease.

Morphologic variation at C6 and C7 was not related to the presence of clinical signs as most OR were <1 for case vs. control. In fact, more morphologic variations were present in the control horses in all cases (A1), spinal ataxia (A2), and in the group with pain on palpation (A3). Only, horses with any lameness (A4) did not differ in having a morphologic variation compared with control horses. The variable age did remain in the final step of case definition A4, leading to the conclusion that the lame horses in the case group were older than the control horses, which were pre‐purchase horses.

## Discussion

This is the first case‐control designed study investigating the possible associations of clinical signs with morphologic variation of the equine cervical vertebral column. The prevalence of morphologic variation at C6 and C7 (28.6%) was similar to a previous study in a more diverse, smaller population of horses 7. Interestingly, these changes were more often seen in the group of Warmblood horses without clinical signs (control) than in the group with clinical signs (cases). Cases defined by lameness showed a similar presence of morphologic variations as control horses. All results suggest no positive relation between morphologic variations and clinical signs as defined in this study.

Homologous morphologic variations at C6 and C7 were found in almost a quarter of the cases and 38% of the controls resulting in an OR>1 for cases vs. controls. The odds in these two populations for having a morphologic variation at C6 and C7 were therefore higher in the group without clinical signs. This is a somewhat unexpected and counter‐intuitive finding, but these results seem to support the fact that caudal cervical vertebral morphologic variation might represent a favourable adaptation in Warmblood horses. However, selection bias of the study population could have influenced these results as horses were presented at different types of clinics (university vs. private equine clinic), for different reasons. The control horses were younger, and potentially more expensive as they were presented for pre‐purchase radiography. This bias was partly controlled in the age and breed restricted analysis, but cannot be excluded entirely. That being said, the prevalence in other studies was close to our observations. A prevalence of 33.3% was recorded in a cross‐sectional post‐mortem CT study 7. A radiographic study 11 found these morphologic variations in 13.3% of a random study population and in 15% (21/138) in a group of Warmblood horses with a higher prevalence in mares. Another cross‐sectional radiographic study described a frequency of 33.5% (19/55) in Warmblood horses without sex association 12, which is again close to our observations. It is possible that geographic, demographic and breed‐related differences can explain the minor differences in frequencies between studies.

Another reason for bias may have been that horses had a different clinical history and reason for presentation. Cases were horses that had been presented at the University clinic because of clinical signs that had been going on for some time. However, controls were pre‐purchase horses without any clinical signs at the moment of examination at the private clinic. No follow‐up data were available of the control horses, and therefore it cannot be ruled out that they developed associated clinical signs later in life. The retrospective aspect of this study and therefore lack of a standardised diagnostic work‐up might also have influenced the outcome. No radiographic association for presence of other vertebral pathologies such as arthrosis was found in cases nor controls, confirming a lack of association as previously reported 12. The multivariable analysis was performed in a subset of horses to equalise groups as it was noted that the case group contained some older horses while the control group contained small numbers of different Warmblood breeds. However, with no plausible reason to be given at this stage, all findings indicated that horses that show clinical signs are less likely to have a congenital morphologic variation of the lower cervical spine, with the exception of cases with lameness. The latter groups were equally likely to show morphologic variation as controls.

## Conclusions

Homologous morphologic variation is common in the caudal cervical spine of Warmbloods. This variation does not appear to be associated with clinical signs and in fact is less frequent in clinical cases than in control animals, perhaps suggesting some protective effect. The existence of such an effect can only be established if the results in this study would be confirmed by other independent prospective studies. These studies should include a standardised clinical work‐up and a well‐documented population of Warmblood horses that needs to be followed over time. The mechanism of any such possible protective effect remains entirely elusive at this stage.

Thus radiographic presence of morphologic variation in the caudal cervical spine must be interpreted with care. There are no indications for any negative effect currently, but its clinical relevance has not yet been fully established and needs further investigation.

## Authors’ declaration of interests

No competing interests have been declared.

## Ethical animal research

Research ethics committee oversight not required by this journal: retrospective analysis of clinical data.

## Owner informed consent

Owners gave consent for their animals' inclusion in the study.

## Sources of funding

None.

## Authorship

S. Veraa contributed to study design, study execution, data analysis and interpretation, and preparation of the manuscript. A.‐J. van den Belt contributed to study design. K. de Graaf, I. Wijnberg and W. Back contributed to study execution. H. Vernooij and M. Nielen contributed to data analysis and interpretation. All authors gave their final approval of the manuscript.

## Supporting information


**Supplementary Item 1:** Univariable analysis in all available horses.

**Supplementary Item 2:** Univariable analysis in a subset of horses <16 years old and being Dutch Warmblood.

**Supplementary Item 3:** Final included variables of multivariable stepwise logistic regression analysis with backward elimination approach in a subset of horses, being <16 years of age and Dutch Warmblood, with shape variation divided in categories.

https://www.podbean.com/eu/pb-rgusw-c968ff

## Manufacturers' addresses

1 Agfa Healthcare, Mortsel, Belgium.

2 VetZ GmbH, Isernhagen, Germany.

3 IBM SPSS Statistics for Windows, Armonk, New York, USA.
