# Supplementary material for: Caudal cervical vertebral morphological variation is not associated with clinical signs in Warmblood horses
Source: Equine Vet J. 2019 Jul 16;52(2):219–24. doi: 10.1111/evj.13140 (PMC7027909; doi:10.1111/evj.13140)
Supplement: Supplementary file 1 — Supplementary Item 1: Univariable analysis in all available horses. [file EVJ-52-219-s001.pdf]

BEVA Equine Veterinary Journal

**Supplementary Item 1:** Univariable analysis in all available horses (*n* = 377). Frequency of variables, odds ratio (OR), 95 % confidence interval (CI) and *p*-value of univariable Pearsons' Chi-square test between horses with clinical signs (case definitions A1: all, A2: spinal ataxia, A3: pain on palpation, A4: overall lameness) and without clinical signs (control). NA = not available and not included in statistical results; DJD = degenerative joint disease; DWB = Dutch Warmblood.

| Variables **ALL** | Categories | Case A1 All (n = 245) N (%) | Case A2 Spinal ataxia (n = 123) n (%) | Case A3 Pain (n = 106) n (%) | Case A4 Lame (n = 116) n (%) | Control (n = 132) n (%) | OR A1 (95% CI) | OR A2 (95% CI) | OR A3 (95% CI) | OR A4 (95% CI) | *p*-value A1 | *p-value A2* | *p-value A3* | *p-value A4* |
|---|---|---|---|---|---|---|---|---|---|---|---|---|---|---|
| Age | NA | 3 (1.2) | 1 (0.8) | 0 (0) | 2 (1.7) | 1 (0.8) | | | | | | | | |
| | 0-10 | 189 (77.1) | 107 (87.0) | 80 (75.5) | 80 (69.0) | 113 (85.6) | 0.6 (0.3-1.0) | 1.1 (0.6-2.4) | 0.5 (0.3-0.95) | 0.4 (0.2-0.7) | 0.055 | 0.733 | 0.034 | 0.002 |
| | >10 | 53 (21.6) | 15 (12.2) | 26 (24.5) | 34 (29.3) | 18 (13.6) | | | | | | | | |
| Sex | NA | 1 (0.4) | 1 (0.8) | 1 (0.9) | 0 (0) | 0 (0) | | | | | | | | |
| | Female | 87 (35.5) | 41 (33.3) | 43 40.6) | 43 (37.1) | 40 (30.3) | 1.3 (0.8-2.0) | 1.2 (0.7-1.97) | 1.6 (0.9-2.7) | 1.4 (0. 8-2.3) | 0.295 | 0.572 | 0.088 | 0.260 |
| | Male | 157 (64.1) | 81 (67.9) | 62 (57.5) | 73 (62.9) | 92 (69.7) | | | | | | | | |
| Breed | DWB | 210 (85.7) | 106 (86.2) | 93 (87.7) | 101 (87.1) | 82 (62.1) | 3.7 (2.2-6.0) | 3.8 (2.0-7.1) | 4.4 (2.2-8.6) | 4.1 (2.2-7.8) | 0.0001 | 0.0001 | 0.0001 | 0.0001 |
| | Other | 35 (14.3) | 17 (13.8) | 13 (12.3) | 15 (12.9) | 50 (37.9) | | | | | | | | |
| DJD | Present | 39 (15.9) | 17 (13.8) | 18 (17.0) | 18 (15.6) | 23 (17.4) | 0.9 (0.5-1.6) | 0.8 (0.4-1.5) | 0.97 (0.5-1.9) | 0.9 (0.4-1.7) | 0.71 | 0.43 | 0.93 | 0.69 |
| | Absent | 206 (84.1) | 106 (86.2) | 88 (83.0) | 98 (84.4) | 109 (82.6) | | | | | | | | |
| Morph. Variation | Yes | 55 (22.4) | 26 (21.1) | 23 (21.7) | 30 (25.9) | 50 (37.9) | 0.5 (0.3-0.8) | 0.4 (0.3-0.8) | 0.5 (0.3-0.8) | 0.6 (0.3-0.99) | 0.001 | 0.003 | 0.007 | 0.043 |
| | None | 190 (77.6) | 97 (78.9) | 83 (78.3) | 86 (74.1) | 82 (62.1) | | | | | | | | |
| | Unilateral | 26 (10.6) | 14 (11.4) | 13 (12.3) | 17 (14.7) | 22 (16.7) | 0.5 (0.2-0.95) | 0.5 (0.3-1.1) | 0.6 (0.3-1.2) | 0.7 (0.4-1.5) | 0.032 | 0.094 | 0.157 | 0.392 |
| | Bilateral | 29 (11.8) | 12 (9.8) | 10 (9.4) | 13 (11.2) | 28 (21.2) | 0.5 (0.3-0.8) | 0.4 (0.2-0.8) | 0.4 (0.2-0.8) | 0.4 (0.2-0.9) | 0.06 | 0.06 | 0.07 | 0.025 |
