# Supplementary material for: Caudal cervical vertebral morphological variation is not associated with clinical signs in Warmblood horses
Source: Equine Vet J. 2019 Jul 16;52(2):219–24. doi: 10.1111/evj.13140 (PMC7027909; doi:10.1111/evj.13140)
Supplement: Supplementary file 2 — Supplementary Item 2: Univariable analysis in a subset of horses <16 years old and being Dutch Warmblood. [file EVJ-52-219-s002.pdf]

BEVA Equine Veterinary Journal

Supplementary Item 2: Univariable analysis in a subset of horses < 16 years old and being Dutch Warmblood (*n* = 290). Frequency of variables, odds ratio (OR), 95 % confidence interval (CI) and *p*-value of univariable Pearsons' Chi-square test between horses with clinical signs (case definitions A1 = all, A2 = spinal ataxia, A3 = pain on palpation, A4 = overall lameness) and without clinical signs (control group). DWB = Dutch Warmblood.

Variables Age<16 & DWB	Categories	Case A1 all (n = 207) n (%)	Case A2 spinal ataxia) (n = 107) n (%)	Case A3 pain (n = 89) n (%)	Case A4 lame (n = 99) n (%)	Control (n = 83) n (%)	OR A1 (95% CI)	OR A2 (95% CI)	OR A3 (95% CI)	OR A4 (95% CI)	*p*-value A1	*p-value A2*	*p-value A3*	*p-value A4*
Age	0-10	167 (80.7)	97 (90.7)	71 (79.8)	70 (70.7)	72 (86.7)	0.6 (0.3-1.3)	1.5 (0.6-3.7)	0.6 (0.3-1.4)	0.4 (0.2-0.8)	0.220	0.394	0.222	0.009
	>10	40 (19.3)	10 (9.3)	18 (20.2)	29 (29.3)	11 (13.3)								
Sex	Female	76 (36.7)	37 (34.6)	37 (41.6)	37 (37.4)	27 (32.5)	1.2 (0.7-2.1)	1.1 (0.6-2.0)	1.5 (0.8-2.8)	1.2 (0.7-2.3)	0.501	0.767	0.220	0.495
	Male	131 (63.3)	70 (65.4)	52 (58.4)	62 (62.6)	56 (67.5)								
DJD	Absent	177 (85.5)	92 (86.0)	74 (83.1)	84 (84.9)	71 (85.6)								
	Present	30 (14.5)	15 (14.0)	15 (16.9)	15 (15.1)	12 (14.4)	1.0 (0.5-2.1)	0.97 (0.4-2.2)	1.2 (0.5-2.7)	1.1 (0.5-2.4)	0.994	0.931	0.666	0.896
Morph. Variation	Yes	49 (23.7)	24 (22.4)	22 (24.7)	25 (25.2)	31 (37.3)	0.5 (0.3-0.9)	0.5 (0.3-0.9)	0.6 (0.3-1.1)	0.6 (0.3-1.1)	0.018	0.025	0.073	0.078
	None	158 (76.3)	83 (77.6)	67 (75.3)	74 (74.8)	52 (62.7)								
	Unilateral	22 (10.7)	13 (12.1)	12 (13.5)	14 (14.1)	14 (16.9)	0.5 (0.3-1.1)	0.6 (0.3-1.3)	0.7 (0.3-1.6)	0.7 (0.3-1.6)	0.077	0.198	0.347	0.398
	Bilateral	27 (13.0)	11 (10.3)	10 (11.2)	11 (11.1)	17 (20.4)	0.5 (0.3-1.0)	0.4 (0.2-0.9)	0.5 (0.2-1.1)	0.455 (0.2-1.1)	0.060	0.031	0.070	0.061
