# Supplementary material for: Caudal cervical vertebral morphological variation is not associated with clinical signs in Warmblood horses
Source: Equine Vet J. 2019 Jul 16;52(2):219–24. doi: 10.1111/evj.13140 (PMC7027909; doi:10.1111/evj.13140)
Supplement: Supplementary file 3 — Supplementary Item 3: Final included variables of multivariable stepwise logistic regression analysis with backward elimination approach in a subset of horses, being <16 years of age and Dutch Warmblood, with shape variation divided in categories. [file EVJ-52-219-s003.pdf]

BEVA Equine Veterinary Journal

Supplementary Item 3: Final included variables of multivariable stepwise logistic regression analysis with backward elimination approach in a subset of horses, being <16 years of age and Dutch Warmblood, with shape variation divided in categories none-yes; for all cases (*n* = 207) or cases defined as horses with pain on palpation (*n* = 89) or lameness (*n* = 99) and the control group (*n* = 82); Sig. = significance, S.E. = standard error, OR = odds ratio; 95% CI = 95% confidence interval.

	Variable	*p*-values (Sig.)	B (S.E.)	OR	95% CI
Case all- control	Morphologic	0.019	-0.654 (0.28)	0.52	0.3-0.9
Case pain on palpation-control	Morphologic Variation	0.075	-0.596 (0.33)	0.6	0.3-1.1
Case lameness - control	Age	0.011	0.998 (0.39)	2.7	1.3-5.9
